# Donor‐derived lymphoma harboring a mosaic sex chromosome in a female stem cell transplantation recipient from a male sibling

**DOI:** 10.1002/jha2.347

**Published:** 2021-12-02

**Authors:** Osamu Imataki, Hiroyuki Kubo, Jun‐ichiro Kida, Makiko Uemura, Haruyuki Fujita, Norimitsu Kadowaki

**Affiliations:** ^1^ Division of Hematology Department of Internal Medicine Faculty of Medicine Kagawa University Kita‐gun Japan

**Keywords:** bone marrow transplantation, chimerism analysis, chromosomal abnormality, stem cell transplantation

**Abbreviations**
CML: chronic myeloid leukemia
CMML: chronic myelomonocytic leukemia
FISH: fluorescence in situ hybridization
SCT: stem cell transplantation

Dear Editor,

We treated a 68‐year‐old woman with malignant lymphoma. She was diagnosed with chronic myeloid leukemia (CML) at the age of 49 years and had undergone stem cell transplantation (SCT). The donor was the patient's 50‐year‐old brother who was healthy at the time of his donation. Human leukocyte antigen loci 8/8 matching confirmed that he was a compatible donor. Before SCT, the patient received a reduced‐intensity regimen of 30 mg/m^2^ fludarabine for 5 days and 3.2 mg/kg IV busulfan for 4 days. The patient did not experience neither acute nor chronic graft‐versus‐host‐disease after transplantation. There was neutrophil recovery on day 23, ascertaining engraftment. Epstein–Barr virus (EBV) antibody status was EB‐VCA IgG × 160, EB‐VCA IgM × 10, and EBNA × 10, before SCT (70 days prior to SCT). After SCT (day 31), there was recovery of EBV antibody status: EB‐VCA IgG × 320, EB‐VCA IgM × 10, and EBNA × 20. She has been on remission from CML for 19 years.

Nineteen years post‐SCT, she visited our institution for persistent abdominal discomfort. An intestinal tumor was identified on computed tomography. She underwent partial resection of the intestine; pathological findings of the resected tumor revealed diffuse large B‐cell lymphoma. The primary lesion formed a mass in the jejunum with invasion of the mesenteric lymph nodes, and the clinical stage was Lugano II. The tumor strongly expressed Epstein–Barr encoding region (EBER), and Ki‐67 was 90% positive pathologically. Fluorescence in situ hybridization (FISH) analysis revealed at least 60% positivity for IgH‐MYC. Both IgH‐BCL2 fusion signal and BCL6 division signal were 0.0%. No infiltration of lymphoma cells was found on bone marrow examination, and chromosomal analysis showed 45,X,‐Y (20/20 cells). Subsequently, the results of a FISH examination of biopsy tissue specimens revealed two types of sex chromosomes: XY, and X (Figure 1A).

**FIGURE 1:**
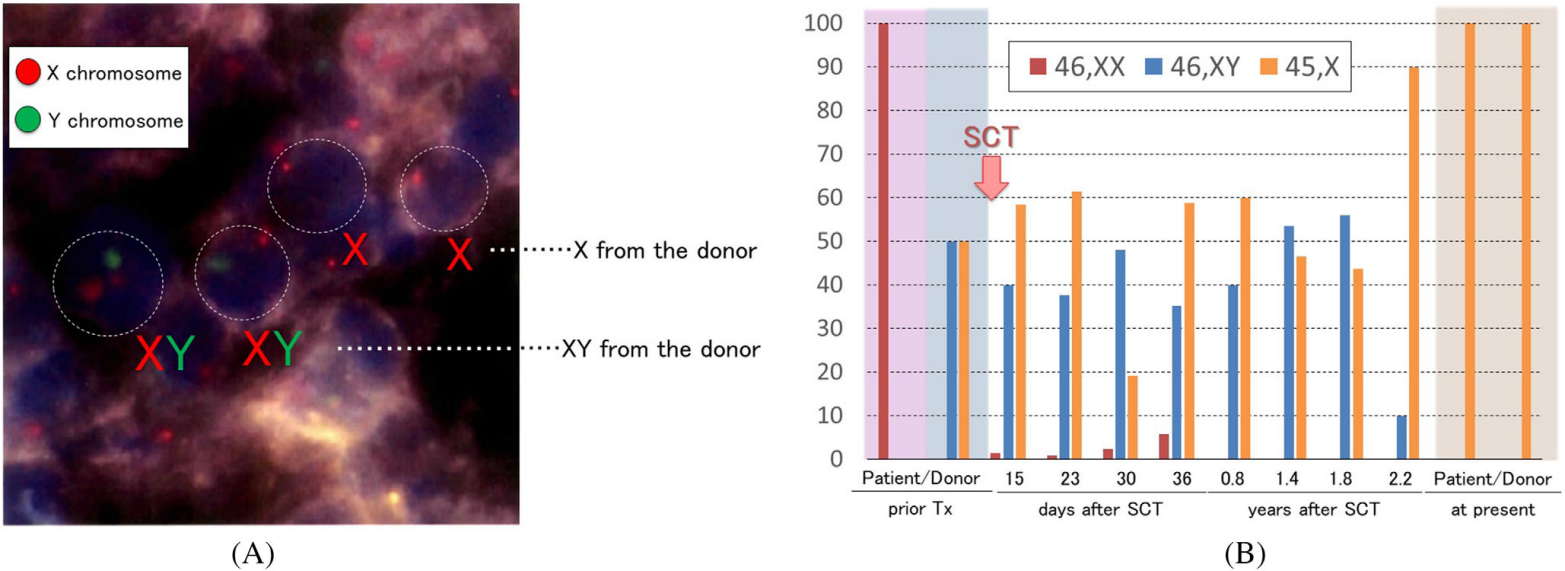
Chromosomal analysis of lymphoma tissue and recipient's and donor's hematopoietic cells. (A) Chromosomal analysis of the recipient's malignant lymphoma 19 years after stem cell transplantation. Fluorescence in situ hybridization (FISH) analysis of the recipient's sex chromosomes in the lymphoma revealed two types of sex chromosomes. Both XY and X signals were morphologically in the large lymphocytes and thought to be donor‐derived. (B) Chromosomal kinetics of the recipient and donor after stem cell transplantation to the present. Retrospectively, we reviewed the recipient's sex chromosome chimerism analysis after stem cell transplantation (SCT). The recipient's sex chromosomes were all 46,XX before transplant, while the donor's sex chromosomes were a mosaic of 46,XY and 45,X,‐Y. The recipient's chromosome pattern shifted to the donor's pattern at the engraftment, and it comprised XY in 40% and Y in 58% of cells. Two years after SCT, the donor's sex chromosomes switched to the donor chimerism of ≥90% 45,X,‐Y. Finally, both the recipient's and the donor's stem cells achieved a complete homozygous donor pattern of 100% 45,X‐Y

Retrospectively, we reviewed the recipient's sex chromosome chimerism analysis after SCT (Figure 1B). Karyotype analysis revealed that the recipient's pretransplant bone marrow cells were 46,XX (20/20), and in the sex chromosome FISH analysis (500 cells), >90% were XX signals. Conversely, the donor's pre‐transplant stem cells comprised 50% 46,XY and 50% 45,X,‐Y. After achieving engraftment, the recipient's hematopoietic stem cells had mixed chimerism comprising recipient‐derived XX in 1.5%, donor‐derived XY in 40%, and Y in 58% of cells. During a 2‐year observation period following SCT, the recipient's stem cells had become a complete chimerism of the donor's mosaic pattern of 46,XY and 45,X,‐Y. After 19 years, the recipient's stem cells achieved complete chimerism of 45,X,‐Y [20/20]. The donor was diagnosed with chronic myelomonocytic leukemia at the age of 69 years, 19 years after his stem cell donation. The present sex chromosomes of the donor are 45,X,‐Y [20/20] (Figure 1B).

Donor‐derived lymphoma is a rare but already known neoplasm that originates from the donor lymphocyte and occurs after SCT [1, 2]. Posttransplant lymphoproliferative disease (PTLD) after SCT is usually donor‐derived, associated with EBV, and of B‐cell origin. This case illustrates the oncogenic time scale of EBV‐associated donor‐derived cells after SCT [3]. The XY chromosome in the recipient existed for only 2 years post‐SCT; therefore, the XY chromosome donor‐derived cells are considered to have been involved in EBV infection within 2 years post‐SCT and progressed to lymphoma after 19 years [4]. There are two types of PTLD: early and late onset. Each PTLD type is characterized according to its EBV status and cytometric phenotype [5]. Treatment outcomes help distinguish one type from the other; however, if EBV infection is associated with its oncogenicity, EBV infection would occur as an early event in the natural history even in late onset‐type EBV‐positive PTLD [5]. Constitutive EBV infections induce T‐cell exhaustion and lead to PTLD [6]. EBV expression is the leading cause of early‐onset PTLD [7]; moreover, PD‐1 expression by continuous stimulation from EBV results in EBER‐positive PTLD [8]. Thus, a typical chronic EBV infection induces T‐cell exhaustion resulting in EBER‐positive early‐onset PTLD; however, this case was EBER‐positive late‐onset PTLD. A recent study identified four major factors influencing the tumor microenvironment in PTLD: EBV infection, chronic antigen stimulation, iatrogenic immunosuppression and donor‐derived immune cells [9]. Any factor can modulate the immunocompetence in the recipient's milieu. This multi‐factorial condition would be associated with the lymphomagenesis of PTLD. Additionally, EBV status is a marker affecting the onset for PTLD. Chronic infection and cancer show a closer relationship [8]; hence, immune‐modulated intervention is warranted to inhibit T‐cell exhaustion, such as anti‐PD‐1/PD‐L1 antibody.

## CONFLICT OF INTEREST

The authors declare no conflict of interest.

## AUTHOR CONTRIBUTIONS

Osamu Imataki managed the patient's case, contributed to the literature search, and wrote the manuscript. Hiroyuki Kubo, Jun‐ichiro Kida, and Haruyuki Fujita made substantial contributions to the concept and design of this report. Makiko Uemura was involved in the supervision of the manuscript. Norimitsu Kadowaki managed the research. All authors approved the final version of the manuscript.

## CONSENT FOR PUBLICATION

Written informed consent was obtained from the patients for publication of this study.

## References

[1] Gandhi MJ , Strong DM . Donor derived malignancy following transplantation: a review. Cell Tissue Bank. 2007;8(4):267–86. 10.1007/s10561-007-9036-1 PMID: 17440834. 17440834

[2] Majhail NS . Secondary cancers following allogeneic haematopoietic cell transplantation in adults. Br J Haematol. 2011;154(3):301–10. 10.1111/j.1365-2141.2011.08756.x. PMID: 21615719. 21615719

[3] Martinez OM , Krams SM . The immune response to Epstein Barr virus and implications for posttransplant lymphoproliferative disorder. Transplantation 2017;101(9):2009–16. 10.1097/TP.0000000000001767 PMID: 28376031; PMCID: PMC5568952. 28376031 PMC5568952

[4] Curtis RE , Travis LB , Rowlings PA , Socié G , Kingma DW , Banks PM , et al. Risk of lymphoproliferative disorders after bone marrow transplantation: a multi‐institutional study. Blood 1999;94(7):2208–16. PMID: 10498590. 10498590

[5] Ghobrial IM , Habermann TM , Macon WR , Ristow KM , Larson TS . Differences between early and late posttransplant lymphoproliferative disorders in solid organ transplant patients: are they two different diseases? Transplantation 2005;79(2):244–7. 10.1097/01.tp.0000144335.39913.5c PMID: 15665775. 15665775

[6] Green MR , Rodig S , Juszczynski P , Ouyang J , Sinha P , O'Donnell E , et al. Constitutive AP‐1 activity and EBV infection induce PD‐L1 in Hodgkin lymphomas and posttransplant lymphoproliferative disorders: implications for targeted therapy. Clin Cancer Res. 2012;18(6):1611–8. 10.1158/1078-0432.CCR-11-1942 PMCID: PMC3321508. 22271878 PMC3321508

[7] Ferreiro JF , Morscio J , Dierickx D , Vandenberghe P , Gheysens O , Verhoef G , et al. EBV‐positive and EBV‐negative posttransplant diffuse large B cell lymphomas have distinct genomic and transcriptomic features. Am J Transplant. 2016;16(2):414–25. 10.1111/ajt.13558 PMID: 26780579. 26780579

[8] Wherry EJ , Kurachi M . Molecular and cellular insights into T cell exhaustion. Nat Rev Immunol. 2015;15(8):486–99. 10.1038/nri3862 PMID: 26205583; PMCID: PMC4889009. 26205583 PMC4889009

[9] Marcelis L , Tousseyn T . The Tumor microenvironment in post‐transplant lymphoproliferative disorders. Cancer Microenviron. 2019;12(1):3–16. 10.1007/s12307-018-00219-5 PMID: 30680693; PMCID: PMC6529504. 30680693 PMC6529504

